# Recent Technologies for Amorphization of Poorly Water-Soluble Drugs

**DOI:** 10.3390/pharmaceutics13081318

**Published:** 2021-08-23

**Authors:** Do-Hyun Kim, Young-Woo Kim, Yee-Yee Tin, Mya-Thet-Paing Soe, Byoung-Hyen Ko, Sun-Jae Park, Jaeh-Wi Lee

**Affiliations:** 1 College of Pharmacy, Chung-Ang University, Seoul 06974, Korea; dylan13@naver.com (D.-H.K.); kimyoungwoo3@naver.com (Y.-W.K.); yeeyeetin18@gmail.com (Y.-Y.T.); mtps.pp@gmail.com (M.-T.-P.S.); badol95@naver.com (B.-H.K.); sunjae1993@naver.com (S.-J.P.); 2 CAU-Myanmar Pacific Pharmaceutical Research Center, Chung-Ang University, Seoul 06974, Korea

**Keywords:** amorphous formulation, solid dispersion, co-amorphous, co-amorphization, in situ amorphization, microwave, mesoporous particles, mesoporous silica

## Abstract

Amorphization technology has been the subject of continuous attention in the pharmaceutical industry, as a means to enhance the solubility of poorly water-soluble drugs. Being in a high energy state, amorphous formulations generally display significantly increased apparent solubility as compared to their crystalline counterparts, which may allow them to generate a supersaturated state in the gastrointestinal tract and in turn, improve the bioavailability. Conventionally, hydrophilic polymers have been used as carriers, in which the amorphous drugs were dispersed and stabilized to form polymeric amorphous solid dispersions. However, the technique had its limitations, some of which include the need for a large number of carriers, the tendency to recrystallize during storage, and the possibility of thermal decomposition of the drug during preparation. Therefore, emerging amorphization technologies have focused on the investigation of novel amorphous-stabilizing carriers and preparation methods that can improve the drug loading and the degree of amorphization. This review highlights the recent pharmaceutical approaches utilizing drug amorphization, such as co-amorphous systems, mesoporous particle-based techniques, and in situ amorphization. Recent updates on these technologies in the last five years are discussed with a focus on their characteristics and commercial potential.

## 1. Introduction

Many developed drugs, approximately 40% of commercially approved drugs, and nearly 90% of drug candidates are poorly soluble in water and thus present low dissolution rate in the gastrointestinal (GI) tract, leading to poor oral bioavailability [1]. Various pharmaceutical technologies have been employed to modify the solubility and dissolution properties of poorly water-soluble drugs, including micronization [2], prodrug formation [3], salt formation [4], microemulsion [5], and amorphization of drugs [6]. Among them, the amorphization method has been considered a highly promising approach, as it directly converts the poorly water-soluble drugs into their amorphous form, thereby markedly enhancing the apparent solubility and the dissolution rate, and as a result, may improve the oral bioavailability of those drugs.

Amorphization is defined as a process that converts a crystalline material into its amorphous state, which can be characterized by the disordered arrangement of drug molecules in the solid state [7]. Crystalline solids are composed of long-range ordered molecules, whereas amorphous solids have short-ranged random molecules. In the process of amorphization, this lack of long-range repetitive structure results in loose packing of molecules and weaker intermolecular attraction forces, and therefore, the drug molecules in amorphous state exhibit higher Gibb’s free energy as compared to those in a crystalline state [8]. Consequently, as less energy is required for the amorphous drugs to go into the “solution state” where the solvent molecules surround the drug molecules, higher apparent solubility, or kinetic solubility, is achieved. In addition, according to the Noyes–Whitney equation, the increase in solubility augments the dissolution rate of the drug [9]. Delivering such solid forms in a high energy state can induce supersaturation of the drug in the GI lumen, and thereby lead to an enhanced intestinal absorption of the drug. Despite these advantages, the main limitation of drugs in an amorphous state is their stability. Due to the high energy state of the amorphous drug particles, they possess a strong tendency to go back to the crystalline state, and thus, are not stable enough to be formulated on their own.

Considering this instability, pharmaceutical scientists have focused on investigating the amorphous-stabilizing strategies, a particular one of which is the amorphous solid dispersion (ASD) technology which utilizes inert carriers (mostly polymers) to disperse the amorphous drugs and prevent their recrystallization [10]. Though the concept of solid dispersions was first suggested in the 1960s, it has since been studied extensively for over half a century and the technology was successfully developed into some commercial products. However, major limitations of the ASD formulations were to be found in the use of hygroscopic polymers and their limited drug solubility [11]. In overcoming these shortcomings, alternative strategies to stabilize the amorphous formulations were required. 

Hence, to successfully develop amorphous drug delivery systems, grasping the concept and history of the solid dispersion technology seems crucial. In this context, the beginning section of this review provides a brief introduction to the conventional amorphous formulations to help readers understand the basic concepts and limitations of the technology and thus, the need for alternative strategies to formulate ASDs. The major aim of this review, however, is to discuss the state of the art in novel approaches employing amorphization such as co-amorphization, mesoporous particle-based amorphization, and in situ amorphization, that were recently reported between 2016 and 2021.

## 2. Solid Dispersion Systems for Amorphization of Poorly Water-Soluble Drugs

As polymers account for a great part of the carriers used, previous works generally used the term solid dispersion somewhat interchangeably with the term polymeric amorphous solid dispersion (PASD). Various pharmaceutical polymers have been explored as carriers to incorporate poorly water-soluble drugs in an amorphous state and protect them from recrystallization. 

In PASDs, drugs are molecularly dispersed in amorphous polymer matrices [12]. Multiple methods have been employed to prepare the PASDs, such as the melting method, solvent evaporation method, and melting-solvent method [12,13,14]. In the melting method, both drugs and polymers are heated and melted together, after which the melt is cooled and solidified rapidly via several methods such as immersion in liquid nitrogen and ice bath agitation. As the melting method does not require the use of organic solvents, the preparation process is considered simple and safe. However, the major disadvantage is that it can only be applied when the drugs and polymers are miscible with each other. Furthermore, the drugs and the polymers should be thermostable because the melting method generally requires high temperatures exceeding the melting points of the components [15,16].

The solvent evaporation method has overcome the intrinsic limitation of the melting method regarding the possible decomposition of drugs due to the need for high temperature settings. In the solvent evaporation method, first, drugs and polymeric carriers are dissolved in solvents, and subsequently, the solvents are removed via various methods such as rotary evaporation, freeze drying, and spray drying. However, as the use of organic solvent may have the risk of residual solvent, the major disadvantage is that the residual solvent can induce physical stability and safety issues [17].

Despite being the most often adopted stabilizing material for ASDs, polymers were shown to have some apparent drawbacks that made commercialization difficult [18]. As the polymers used in PASDs are hygroscopic in nature, they tend to absorb moisture, which can increase the recrystallization potential of the incorporated drug even at room temperature. In addition, because of the limited solubility of drugs in the polymers, large amounts of polymers are required to maintain the stability of the amorphous drugs, which in turn increases the total volume of the dosage form. The physicochemical stability of polymer-based ASD has been affected primarily by polymer viscosity, the weight fraction of the polymer, and the polymer selected choice of solvent in the spray-drying process. For instance, the stability of acetaminophen dispersed in polyvinylpyrrolidone (PVP) was best with PVP K17, the least viscous PVP among the PVPs tested [19].

## 3. Emerging Pharmaceutical Techniques for Amorphization of Poorly Water-Soluble Drugs 

**Figure 1:**
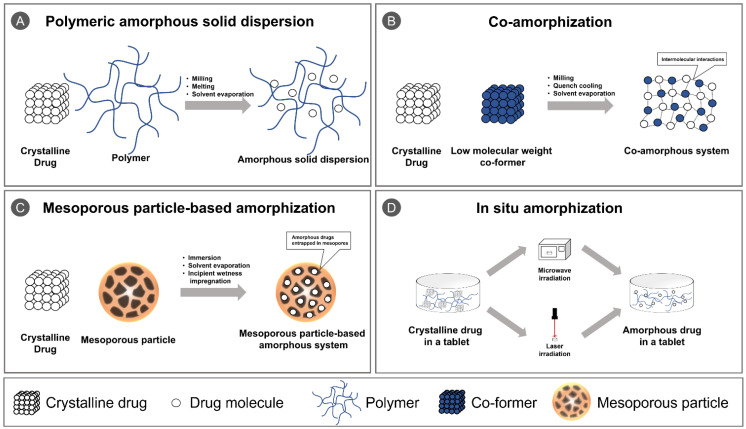
Schematic illustration of (**A**) polymeric amorphous solid dispersion; (**B**) co-amorphization; (**C**) mesoporous particle-based amorphization; and (**D**) in situ amorphization.

In various attempts to overcome the limitations of PASDs, novel techniques such as co-amorphization, mesoporous particle-based amorphization, and in situ amorphization have been introduced (Figure 1). There are several reviews that focused on each approach, but to the best of our knowledge, only a few have done comprehensive reviews on these technologies, and they lack recent updates on the attempts that are being made. Therefore, in the subsequent sections, the basic concept of the different technologies, their advantages and disadvantages, the future perspective of these technologies, and the recent research trends of the last five years will be discussed.

### 3.1. Co-Amorphization Techniques

#### 3.1.1. Concept of Co-Amorphization

Co-amorphous systems have emerged as an alternative approach to the conventional PASDs, which have been extensively studied in this field for decades [20]. The term ‘co-amorphous’ was introduced by Chieng et al. (2009) to differentiate the amorphous systems composed of substances with a low molecular weight from the PASDs [21]. The co-amorphous system is defined as a single-phase homogeneous amorphous system that comprises multiple components of a low molecular weight instead of using polymers which have a high molecular weight. In co-amorphous systems, several mechanisms are involved in the stabilization of the amorphous drugs such as an increase in T_g_ of the mixture, which also appears in the formation of PASDs [22]. In fact, a number of studies in co-amorphization technologies have ascribed the stabilization of drugs in an amorphous state to intermolecular interactions between the components, including hydrogen bonding and π-π interactions [21,23,24,25]. Compared to PASDs, co-amorphous systems have a higher degree of intermolecular interactions between drugs and small molecular carriers, and therefore recrystallization, one of the major limitations in PASDs, can occur less frequently. In addition, co-amorphous SD systems present high conformational flexibility and availability of molecular level mixing, which enable the components to have better miscibility with one another as compared to those in PASDs [26,27]. 

#### 3.1.2. Preparation Methods for Co-Amorphous Systems

Various methods were employed to prepare co-amorphous systems (Table 1), and the preparation methods for co-amorphization can be classified into three different types: milling, quench cooling, and solvent evaporation [20]. The milling technique, which utilizes mechanical activation to convert materials into an amorphous state, is the most commonly used method due to its convenience of handling [28,29,30]. It also achieves less chemical degradation and exhibits relatively high yield recovery. Despite these advantages, it requires a great deal of time and effort and is likely to leave crystalline impurities which could be a contaminant to the mixture. There are two different types of milling: ball milling and cryo-milling. Cryo-milling differs from the conventional milling in that the process is conducted in cryogenic conditions, where liquid nitrogen is introduced to milling bowls and grinding spheres [29]. As the milling temperature is important in the transition, by keeping the temperature below T_g_, the formation of amorphous state by mechanical activation becomes relatively easier. For these reasons, cryo-milling could be considered more efficient for the production of co-amorphous formulations. 

In the process of quench cooling, the drug mixture is heated first, then the molten drug is rapidly cooled. The cooling time is kept short enough to hinder molecules from rearranging into crystalline lattice, thereby making it amorphous [31]. Quench cooling is useful for producing small-quantity ASD formulations, particularly for the thermostable drugs. However, some drawbacks to this technique include possible chemical degradation of the materials and the need for strict control of the freezing rate.

In the solvent evaporation method, co-amorphous systems are fabricated in a similar manner to PASDs. Several recent works studied the influence of preparation methods in the formation of co-amorphous formulations (Table 1) [30,32], and one of them demonstrated the use of the spray drying technique to successfully prepare the co-amorphous systems of the basic drug carvedilol and the acidic amino acids glutamic acid and aspartic acid [30]. 

Recently, supercritical fluid technology, utilized in fabricating PASDs, was successfully applied to prepare co-amorphous systems [32]. A supercritical fluid is referred to as a substance at a pressure and temperature over its critical point, and it has the advantages of both being both a liquid and a gas. The supercritical carbon dioxide has been mostly utilized as a solvent for dissolving drugs and co-formers, or an antisolvent to precipitate co-amorphous particles. Park et al. produced a glimepiride-L-arginine co-amorphous system by employing supercritical carbon dioxide as an antisolvent [32]. They demonstrated that the supercritical antisolvent process could produce a pure co-amorphous system in terms of the crystallinity, compared to physical mixing and the solvent evaporation method. Furthermore, chemical decomposition of the drug was not observed in the supercritical antisolvent process, while the melt quenching method caused a decrease in the drug content owing to decomposition of the drug.

**Table 1:** Recent publications that employed various methods to prepare co-amorphous systems.

Drug	Carrier	Preparation Method	Key Findings	Ref.
Indomethacin Mefenamic acid	Meglumine Polyvinylpyrrolidone	Co-milling	The glass forming ability of a drug has a major effect on the dissolution of its milled and co-milled compositions. Co-amorphous formulations with meglumine achieved a ten-fold higher drug release than those with polyvinylpyrrolidone.	[28]
Hydrochlorothiazide	Atenolol	Cryo-milling	Cryogenic milling, the solvent-free method, was a promising technique to fabricate the hydrochlorothiazide-atenolol co-amorphous system.	[29]
Carvedilol	Glutamic acid Aspartic acid	Ball milling Spray drying Liquid-assisted grinding	Spray drying was the only successful method for preparing complete co-amorphous systems. The presence of solvents was not key in selecting the preparation technique.	[30]
Glimepiride	Arginine	Physical mixing Melt quenching Solvent evaporation Supercritical antisolvent process	The supercritical antisolvent process turned out to be most suitable for producing Glimepiride-Arginine Co-amorphous formulations.	[32]

#### 3.1.3. Drug-Drug and Drug-Excipient Co-Amorphous Systems

Co-amorphous systems can be classified into two main categories on the basis of their components as drug-drug mixtures and drug-excipient combinations [20]. A drug-drug co-amorphous system is a binary amorphous system where pharmacologically relevant drugs are combined and stably maintain each other in the amorphous state [33,34]. As each small molecular drug in the formulation plays both roles as a stabilizing agent and an active pharmaceutical agent, co-amorphous systems could be formed with only small amounts of ingredients, thus leading to a decrease in the total amount of drugs to be administered. In addition, a multidrug formulation could be efficiently designed because it combines two drugs in different therapeutic categories. 

However, as it is difficult to find two different drugs which are pharmacologically relevant and have glass forming capability, the application of drug-drug co-amorphous systems is limited. Furthermore, the therapeutic advantage is not clear without providing supporting-data regarding drug-drug interactions, and there are also regulatory issues for commercially developing the multidrug formulations. These limitations, therefore, led to the development of co-amorphous systems fabricated via combining drugs with other small molecular excipients, for example organic acids and amino acids, which can present adequate intermolecular interactions with the drugs.

In drug-excipient combinations, the excipients with a small molecular weight, generally called co-formers in this field, act as stabilizers in co-amorphization to enhance the physical stability and dissolution rate of drugs [33,34,35,36,37,38,39,40,41,42,43,44,45]. The selection of an adequate conformer which can form sufficient intermolecular interactions with the drug, such as charge-assisted interactions and hydrogen bonding, is therefore an important step. Various co-formers that have been employed include amino acids, a multifunctional carboxylic acid or organic acids, nicotinamide, folate, bile acid, bioflavonoids, and sugars. 

##### Co-former Selection for Co-Amorphous Systems

So far, there have been extensive efforts to find adequate co-formers capable of forming co-amorphous systems with drugs and the optimal ratios between them (Table 2). Amino acids as co-formers have been particularly well studied in this context. Kasten et al. investigated the co-formability between six drugs and twenty amino acids, which can provide 120 co-amorphous combinations, and these results can be utilized as a guide to search successful co-amorphous formulations containing amino acids [37]. Subsequently, they further studied not only the co-formability but also the physical stability of the 36 successful co-amorphous drug-amino acid, which are indispensable aspects in this field, and collectively, suggested the guidelines for screening amino acids as co-formers for the formulation of co-amorphous systems with new drugs [38].

Dipeptides, which are composed of two amino acids, were also attempted to be utilized as a co-former where rationales were that dipeptides might present the inherent characteristics of both amino acids [39]. As dipeptides consist of two amino acids, the physicochemical properties can be modified through combining different amino acids with various side chains and differing sequences. In this study, the co-amorphous systems were successfully formed via using five dipeptides having different properties as co-formers to stabilize the hydrophobic model drug mebendazole.

Along with amino acids, organic acids as co-formers have been explored, such as tannic acid, benzoic acid, malic acid, and citric acid [41,44,45]. Wu et al. studied the potential of three organic acids, including benzoic acid, malic acid, and citric acid, as co-formers combined with carvedilol, and further found the optimal molar ratio between the organic acids and the drugs in the production of co-amorphous systems [44]. Another study found that tannic acid could be used as a co-former to fabricate co-amorphous systems with carbamazepine and indomethacin by successfully forming intermolecular interactions [45]. From these studies, the potential of organic acids as co-formers was demonstrated. Furthermore, as there are large number of organic acids available, they offer multiple options for the selection of co-formers when formulating co-amorphous SD systems.

#### 3.1.4. Predictive Evaluation of the Formation of Co-Amorphous Systems

As understanding the specific mechanism for the formation of co-amorphous systems is vital, several researchers have addressed this issue [46,47,48,49,50,51]. Table 3 shows the summary of recent publications on the prediction of co-amorphous formation. Kissi et al. suggested using the glass-transition temperature for the β-relaxation as the parameter that can predict the potential of the successful formation of co-amorphous systems [46]. The conditions required for the formation of the co-amorphous systems, however, have not been clearly provided. Following this, Mizoguchi et al. demonstrated the method to predict the compounds that can be used to form co-amorphous systems via utilizing two parameters entailing mixing enthalpy (ΔH_mix_) and Δlog *p* values [49]. They found that the chances to form co-amorphous systems are high when the mixtures present negative ΔH_mix_ and similar lipophilicity (small Δlog *p* values). In addition, prediction of the physical stability was conducted. The physical stability was found to be high when co-amorphous formulations are composed of co-former constituents with stable amorphous state and show negative ΔH_mix_ values. Meng-Lund et al. fabricated 120 co-amorphous systems combining 6 representative drugs and 20 amino acids [50], which seems similar to the study done by Kasten et al. [37], but different aspects were reflected in that they investigated the molecular descriptors representing the properties of the combinations such as lipophilicity, surface area, volume, and pKa. Collectively, these recent studies would be useful for the researchers to understand the mechanism and even predict the possibility of the formation of the co-amorphous systems, and the extent of physical stability.

#### 3.1.5. Applications of Co-Amorphous Systems in Different Routes of Administration

Co-amorphous systems have generally been developed as oral formulations. However, several researchers have recently focused on the design of co-amorphous systems which can be administered by other routes, such as the transdermal route, the parenteral route, and the inhalation route, instead of the oral route (Table 4). The applicability of co-amorphous systems in the transdermal delivery was investigated by generating a supersaturated co-amorphous formulation comprising atenolol and urea as the model drug with a low permeability and the co-former, respectively [52]. This supersaturated formulation maintained its inhibition effect of recrystallization for about two months and could largely enhance the in vitro skin permeation of the drug, even compared to the permeability predicted from the degree of supersaturation. 

Parenteral route was also explored to deliver co-amorphous formulations of zwitterionic compounds with amino acids [53]. This co-amorphous drug formulations for parenteral delivery could present more than a 10-fold increase in the apparent solubility of ofloxacin, a model drug with a zwitterionic nature, through combining the drug with tryptophan, an amino acid utilized as the co-former in this study. A complete amorphization of the drug with tryptophan was demonstrated through X-ray diffraction analysis. Due to the low molecular weight of tryptophan and the high solubilizing capacity of the co-amorphous drug formulation, the injection volume for a certain dose could be significantly reduced, and these appealing characteristics were advantageous for parenteral delivery systems. 

Lu et al. designed a spray-dried co-amorphous system for inhalation [54]. They demonstrated that spray drying could be readily exploited to produce co-amorphous inhalable dry powders containing budesonide and arginine as a drug and co-former, respectively. Indeed, in the stability test of 210-day storage at room temperature, these inhalable co-amorphous dry powders exhibited higher physical stability than spray-dried budesonide alone. A similar study from the same laboratory focused on the effects of various amino acids to fabricate co-amorphous inhalable dry powders using simvastatin as a model drug [55]. The results suggested that the effects of amino acids on the stability and aerodynamic performance of co-amorphous dry powders were not greatly influenced by the specific nature of amino acids, such as hydrophilic and hydrophobic properties, but instead depended on the pairs of drugs and amino acids. Among the three different amino acids (leucine, tryptophan, and lysine) used in this study, leucine was the best excipient for fabricating co-amorphous dry powders with simvastatin in terms of physical stability and aerodynamic performance. 

### 3.2. Mesoporous Particle-Based Amorphization

#### 3.2.1. Concept of Mesoporous Particle-Based Amorphization

Mesoporous particles have recently gained considerable attention as a stabilizer for amorphous formulations. The mesoporous particles refer to the porous particles with pore diameters in the range of 2 nm and 50 nm, while other types of porous particles such as microporous and macroporous particles show pore diameters as smaller than 2 nm and larger than 50 nm, respectively. The majority of the mesoporous particles utilized in the pharmaceutical field, are mesoporous silica nanoparticles such as MCM-41, Neusilin^®^ UFL2, Fujicalin^®^, and Fujisil^TM^-F. These mesoporous silicas are generally produced by electrochemical techniques, including stain etching and anodization [56]. Originally, they have been used as adsorbents to fabricate solid dosage forms since the 1970s. In 2001, Vallet-Regi et al. newly suggested the use of mesoporous silica MCM-41 in a drug delivery system that can carry drug molecules in the pore structure [57]. Following this, the mesoporous particles have been intensively studied to progress this idea with varying concepts. 

Several mesoporous silica nanoparticles available on the market have different properties such as their pore size, pore volume, and surface area [58]. Their large pore volume and surface area allow them to incorporate large amounts of therapeutic agents. In fact, their characteristic pore structure has been reported to entrap drug molecules in an amorphous state and prevent the drug from recrystallizing, thereby acting as the stabilizer for amorphous formulations. In addition, due to silanol groups on the surface of the mesoporous silica, drug-silica interactions may occur mainly via hydrogen bonding, thereby leading to the amorphization of the drug molecules. 

#### 3.2.2. Drug Loading and Amorphization Methods for Mesoporous Particle-Based Amorphization

Drug loading and amorphization process of the mesoporous particles is different from those of other ASD systems. The particle fabrication process and drug loading process to the mesoporous particles are separated, and as such, the drug loading capacity in the mesoporous particles is inherently lower than other solid carriers, including hydrophilic polymers used in PASDs. In order to address this issue, multiple loading techniques have been suggested to efficiently entrap a drug into the pores of the mesoporous particles. In this section, therefore, the conventional methods are briefly summarized as they are still widely utilized to this day, and the recent attempts involving this topic will be discussed in particular. Table 5 summarizes the most up-to-date research articles covering the drug loading and amorphization methods for mesoporous particle-based amorphization. 

##### Solvent-Free Methods

Drug loading methods for the mesoporous particle-based systems can be classified under two large groups: the solvent methods and the solvent-free methods. Despite the solvent methods being the most often adopted methods, solvent-free methods such as solid-state reaction and fluid-bed hot-melt impregnation are still being used and are likely to advance currently [63]. The solid-sate reaction method generally utilizes a ball mill to mix the drug with mesoporous particles on the basis of the interaction between drugs and the silanol groups on the surface of the particles. Although this method was not able to ensure the complete adsorption of drugs in the pores, recent work reported that the solid-state method was more efficient for curcumin loading and amorphization in the pores than the incipient wetness impregnation method as one of the solvent methods [64].

One of the solvent free methods, the melting method is carried out by mixing the drug with the mesoporous particles, followed by heating the mixture above the melting point to generate the molten drug to be loaded in the mesopores. Mužík et al. demonstrated the potential of the fluid bed hot-melt impregnation method carried out through hot-melt loading of drugs into the mesoporous particles in a fluidized bed device [63]. In this work, the fluid bed hot-melt impregnation method achieved high drug loading over 30%, showing a crystallinity decrease and indicating that the drugs are loaded within the pores in amorphous forms. As the fluidized bed device could offer many advantages such as an easy scale-up and good reproducibility of the product, this fluid bed hot-melt impregnation method was considered to be a more efficient technique compared to the conventional melting method.

##### Solvent Methods

The main principle for drug loading using solvents is capillary action, where drug solution or slurry permeate the pores, followed by the adsorption on the surface of the pores [65,66]. As for the immersion method, the drug is dissolved in a solvent, generally organic solvent, and mesoporous particles are added to the drug solution. The solvent is then removed by filtration, and the drug-loaded mesoporous particles are further washed several times to eliminate excess drug which could be adsorbed on the surface instead of the pores of the mesoporous particles. Additionally, thermogravimetric analysis can be used to differentiate the drug adsorbed within the pores and on the surface of mesoporous particles by comparing their transition profiles [64,67].

As the drug loading into the mesoporous particles is limited by the pore volume, there is actual maximum amount to be loaded. However, the drug loading via the immersion method is far lower than this maximum amount, meaning that the drug cannot be fully adsorbed in the pores. Several reasons have been suggested for this low drug loading. Simple immersion of the particle in the drug solution is not enough to provide the sufficient capillary force to load the drugs deeply into the pores. Furthermore, during filtration, centrifugation, and washing, the drugs incorporated in the pores can be washed away, leading to low drug loading.

The solvent evaporation method is quite similar to the immersion method [68]. One major difference is that the solvent is removed by a vacuum evaporation process instead of the filtration step utilized in the immersion method, and this enables a significant reduction in the drug loss. In addition, the solvent evaporation process inherently offers a driving concentration gradient between drug and mesoporous particles because the drug concentration in the solution increases during the process. The drug loading via the solvent evaporation method is therefore more efficient than the immersion method.

Regarding the incipient wetness method, a concentrated drug solution was prepared and subsequently added onto the mesoporous particles in a dropwise manner [64]. The drug solution added onto the particles permeates into the pores through a capillary action, and the solvent with a small volume can be rapidly evaporated. After repeating this process, the drug-adsorbed mesoporous particles are obtained, and the washing process is generally conducted to ensure that the loaded drugs are in the pores. As this method considerably reduces the loss of drugs thereby leading to high drug loading, it is useful for expensive drugs. 

However, for the solvent-based methods, the use of organic solvents has been the main issue to be addressed because of the associated physical stability and safety problems. The supercritical fluid technology was therefore applied to load drugs onto the mesoporous particles via utilizing the supercritical carbon dioxide as a solvent for dissolving drugs instead of organic solvent [59,60,67]. Wang et al. have compared the supercritical fluid technique with conventional processes such as the immersion method in terms of drug loading capacity [67]. It was found that the supercritical fluid technique was the most efficient in increasing drug loading due largely to rapid evaporation of supercritical carbon dioxide following the penetration of a drug solution into the pores and pressures applied. In the similar study from a different laboratory [60], the supercritical fluid method showed approximately a 1.6-fold higher fenofibrate loading capacity and 24-fold faster loading time into mesoporous particles over the incipient wetness method.

##### One Pot Synthesis and Drug Loading Method

To overcome the inherent limitation in low drug loading due to the separated process of the particle synthesis and the drug loading, one-pot synthesis method was suggested where the drug loading process was simultaneously conducted during the particle synthesis. The one-pot synthesis method generally utilizes surfactants that have been employed upon fabricating the mesoporous particle. The drugs are incorporated in the micelle, after which the silica source is added to form the structure of the mesoporous particles. Tourne-Peteilh et al. demonstrated the potential of a sol-gel one-pot synthesis method which utilizes Tween^®^ 80 as a solubilizing agent for a hydrophobic drug and simultaneously as a template for the particle synthesis under ambient conditions and without using organic solvent [69]. However, the drug loading was still shown to be low, and also several days were required for this method.

As a similar study utilizing one-pot synthesis method, Wan et al. applied the evaporation-induced self-assembly technique in the one-pot synthesis process upon loading a hydrophilic drug heparin or hydrophobic drug ibuprofen into a mesoporous silica SBA-15 [62]. This technique could provide several-fold higher drug loading compared to conventional loading methods like immersion. The preparation method was also reduced from 74 h to 10 h. As considerably different results on drug loading via the one-pot synthesis method were found between the previous works, we can realize that various experimental conditions are crucial for this method and this technique actually continues to advance.

#### 3.2.3. Surface Modification Strategies for Mesoporous Particle-Based Amorphization

As in other studies on the microparticle-based drug delivery systems, pharmaceutical scientists have attempted to modify the surface on the mesoporous particles to improve various properties, such as drug loading, targeting effect, and stability. The surface modification strategies have been the subject of many studies on mesoporous particle-based amorphization for about 20 years, and Table 6 shows the recent publications on this topic. The aim of this section is hence to present the most up-to-date papers on this subject with a focus on distinct characteristics of their technologies, and the rationale behind their works, which can be helpful for the pharmaceutical scientists to further study this topic.

Ayad et al. introduced amine modification to the surface of mesoporous silica to improve the loading capacity of ketoprofen and 5-flurouracil, and also control the drug release from the mesoporous system [70]. The data on drug loading capacity revealed that the amino-modified mesoporous silica achieved a four-fold increase in the drug loading compared to the unmodified counterpart. Also, the amino surface modification allowed a pH-responsive release pattern for the drug, and therefore more drugs could be delivered in a controlled manner.

Szegedi et al. developed amino-modified mesoporous particles coated with polyelectrolyte polymer complex [64]. The amino groups-modified mesoporous particles were fabricated, after which curcumin was loaded in the particles via incipient wetness impregnation or a solid-state reaction. After that, the drug-loaded mesoporous particles were coated by two polymers showing opposite charges with κ-carrageenan having a negative charge and chitosan, a positive charge. The oppositely charged polymers were alternately deposited on the surface of the particles, forming multi-layered polymer coatings around the particles. The surface-modified mesoporous particles considerably improved the curcumin solubility and the dissolution, probably due to the amorphization of the drug as proved by X-ray diffraction analysis. In addition, the multi-layered coatings, composed of κ-carrageenan and chitosan, were able to control the release of the drug from the particle and improved the drug stability.

Narayan et al. designed a chitosan-glucuronic acid conjugate-coated mesoporous particle for the efficient delivery of capecitabine to the colon [66]. Capecitabine, the first-line drug for colorectal cancer, was loaded into the pores of the mesoporous particles via immersion and the solvent evaporation method, after which the drug-loaded mesoporous particles were coated with chitosan-glucuronic acid conjugate. Due to the pH-responsive property of chitosan, it could offer pH-responsive release of the drug from the mesoporous particle at the slightly acidic extracellular condition of the solid tumors. In addition, the glucuronic acid conjugation to the particle could promote receptor-targeted delivery of the formulation, as glycosylated formulation can preferentially bind to the lectin receptor, generally overexpressed on the surface of the tumor cells, and thereby lead to the uptake increase. Furthermore, to prevent the drug release before reaching the colon once orally administered, Eudragit^®^ S-100 was additionally used to coat the chitosan-glucuronic acid conjugate mesoporous particles. Overall, the formulation developed in this study achieved specific and targeted release of the drug to the colorectal tumor. 

Folic acid and methionine were employed for the surface modification of mesoporous particles to offer tumor targeting effect to the particles [71,72]. As tumor cells have overexpressed folate receptors and require much more methionine compared to normal cells, researchers have attempted to improve tumor cell uptake of the formulations through combining folic acid/methionine with the mesoporous particles. Khosravian et al. designed mesoporous particles functionalized with folic acid or methionine for targeted delivery of docetaxel for breast cancer [71]. Amine-functionalized mesoporous nanoparticle was first prepared, and its surface was further modified with folic acid or methionine. The mesoporous particles functionalized with folic acid or methionine achieved several-fold higher intracellular uptake over the unmodified counterpart.

**Table 6:** Recent examples of surface modification strategies for mesoporous particle-based amorphization.

Drug	Carrier	Preparation Method	Key Findings	Ref.
Curcumin	Mesoporous silica synthesized in the laboratory	Incipient wetness impregnation method Solid-state reaction method	Multi-layered coatings with two polymers showing opposite charges, κ-carrageenan and chitosan, could control the drug release from the mesoporous particle and improve the drug stability.	[64]
Capecitabine	Mesoporous silica synthesized in the laboratory	Immersion method Solvent evaporation method	Chitosan coatings could offer pH-responsive release of the drug from the silica. Glucuronic acid conjugation to the particle could promote receptor targeted delivery to the tumor cell.	[66]
Docetaxel	Mesoporous silica synthesized in the laboratory	Immersion method	Mesoporous particles functionalized with folic acid or methionine achieved a several-fold higher intracellular uptake to the breast cancer cells over the unmodified counterpart.	[71]
Cis-Pt	Mesoporous silica synthesized in the laboratory	Immersion method	Folic acid modification on the mesoporous particle surface could offer active targeted delivery of Cis-Pt to glioblastoma cells.	[72]
Fluorouracil	Mesoporous silica synthesized in the laboratory	Immersion method	A magnetic and temperature responsive drug delivery system was designed via fabricating the nanocomposite consisting of iron oxide as core, silica as middle layer, and poly(N-isopropyl acrylamide-co-acrylic acid) as an exterior layer.	[73]

### 3.3. In Situ Amorphization

#### 3.3.1. Concept of In Situ Amorphization

The concept of in situ amorphization has caught the attention of many researchers seeking new ways to deal with the physical stability problem of amorphous formulations. It was first seen as more of an undesired anomaly or a deviation in the manufacturing process rather than a different and novel approach to produce amorphous products. The spontaneous and unintended amorphization was observed with different combinations of drugs and polymers in various settings [74]. However, what these cases had in common was that either moisture or physical stimulation, during storage or the manufacturing process, was involved. Once they discovered that such factors were at play, researchers sought ways to “induce” or even accelerate the pace of this so-called undesired loss of crystallinity. The idea was that if a drug prepared as a crystalline mixture can be amorphized on-demand, moments before the administration, the possibility of recrystallization taking place in the long term during storage can be avoided. Some of the initial attempts had been focused mainly on preparing the amorphous formulations in the bulk powder form and we suggest the readers to refer to the review article by Qiang et al. for further detail [75]. In this review, we intend to highlight the application of in situ amorphization in the final dosage form: tablets. Table 7 summarizes some of the previous, as well as up-to-date research articles covering the in situ amorphization within the tablet formulations. 

#### 3.3.2. Different Methods for In Situ Amorphization

##### Solvent-Assisted Method

The first amorphization attempt within the tablet was done through immersion in an aqueous buffer (pH 6.8) [76]. Eudragit^®^ E, the polymeric carrier used, was plasticized at pH 6.8 and dissolved at pH 4.1, releasing the amorphous drug. In a subsequent study by Doreth et al., Eudragit^®^ E was compacted with different active pharmaceutical ingredients (APIs), and the tablet was immersed in water to be amorphized. The authors suggested that this on-demand amorphization could be done through simply placing the tablet in a glass of water for a few minutes [77]. 

Petry et al. demonstrated the feasibility of in situ co-amorphization within the tablet formulation. The authors prepared compacts comprising indomethacin and arginine, a combination previously shown to generate co-amorphous systems, and immersed them in an acidic medium as an in vitro model of the gastric fluid [81]. In a previous study by Petry et al., the co-amorphization of indomethacin and arginine was observed in a storage setting of 75% relative humidity. The authors suggested that the formation of arginine dihydrates may have facilitated the interactions between the drug and the amino acid [91]. It seems that the ordered structure of the crystalline drug was interfered with by such interactions, and at higher concentrations of the amino acid co-former, the co-amorphous system was generated. The applicability of in situ co-amorphization was further demonstrated when another co-amorphous formulation was successfully prepared in the final dosage form, using carvedilol and aspartic acid [82]. These results may suggest the possibility of an in vivo amorphization model, in which drugs can be amorphized in the GI tract after the administration.

##### Microwave Irradiation

Microwave irradiation has been used in the pharmaceutical industry, mostly for drying purposes [92]. When subjected to microwave radiation, dipoles in the sample oscillate rapidly and as a result, produce heat. Also, as the radiation penetrates and works internally, heating by a microwave oven is known to be more homogeneous as compared to that of the convection oven [84]. This type of rapid and homogeneous heating provided by such an easily accessible tool was, thus, considered as an attractive option in the production of amorphous systems. 

Doreth et al. were the first to employ microwave irradiation to amorphize the final dosage form [78]. Tablets containing the physical mixture of indomethacin, PVP K12, and lubricant were prepared by direct compression. Subsequently, the compacts were conditioned in an environment with a certain level of relative humidity for the polymers to uptake water, as it is required to absorb microwave radiation. Upon exposure to the microwave radiation, indomethacin, a model drug known to change color (white to yellow) as it becomes amorphous, turned yellow and this change in crystallinity was confirmed through X-ray diffraction analysis and differential scanning calorimetry. The major objective of the study was to observe the effect of water content and energy input in the in situ amorphization process. By varying the relative humidity settings, it was demonstrated that with higher humidity conditioning, the degree of amorphization was increased.

In a subsequent study, the authors explored the relationship between the T_g_ of the polymer and the degree of amorphization induced by microwave irradiation [79]. Amongst the three types of PVPs with differing molecular weights, PVP K12 showed the lowest molecular weight, and thus T_g_, was able to achieve the highest degree of amorphization. From these results, it was assumed that heating the samples above their T_g_ for a certain duration was necessary to alter the crystallinity of the drug. In these studies, however, none of the resultant formulations were fully amorphous. 

Based on the hypothesis that this limited amorphicity may be related to the drug load in the system, Edinger et al. investigated how the change in drug load affects the relative amorphous fraction of the API using Transmission Raman Spectroscopy. It was shown that with a lower drug content, a higher amorphous fraction (though still not 100%) was obtained [80]. Controlling other factors, e.g., crystal size, seemed to be required to produce a fully amorphous formulation in situ. In 2020, by reducing the particle size of the drug and polymer, the first case of fully amorphous compact prepared in situ by microwave irradiation was reported [83].

In these settings involvement of water was a necessity to produce glass solutions in situ by microwave irradiation due to its function as a plasticizer and a microwave absorber. However, this reliance on water had to be dealt with, given its well-known ability to cause recrystallization and possibly chemical degradation of the drug, not to mention the inconvenience of the moisture-conditioning step itself [85]. In light of this, authors started to look towards alternative sources that absorb microwave radiation and at the same time, work as a plasticizer for the polymers. 

Glycerol was used as a microwave absorbing entity due to its dielectric properties and plasticizing function [85]. In another approach, hydrate forms (mono- and dihydrate) of sodium dihydrogen phosphate were successfully utilized as the alternative source of water, allowing the pre-conditioning step to be circumvented [87]. In majority of cases, PVP, which is practically unable to absorb microwaves on its own, was the polymer of choice. If the polymer itself can be the microwave absorber, moisture sorption is no longer a prerequisite. Polyethylene glycol, which was reported to absorb a certain amount of microwave radiation in temperatures above its melting point, was tested for this purpose and has effectively shown its potential as a carrier for the in situ amorphization of celecoxib without any moisture conditioning [86,89].

##### Laser Irradiation

As the microwave irradiation method necessitated the involvement of a microwave absorbing plasticizer (water), which carries the risk of causing hydrolysis of the API, there seemed to be a need for a new method to fabricate amorphous systems in situ. In a recent study by Hempel et al., laser irradiation was selected as an alternative heating source with photothermal plasmonic nanoparticles (PNs) as the laser absorbing component. PNs are stimuli-responsive materials that can be activated by laser at their plasmonic resonance frequency and release vibrational energy in the form of heat. Compacts containing the physical mixture of celecoxib, PVP, and plasmonic silver nanoaggregates were prepared with different drug loads (30, 50 wt %) and PN concentrations (0.1, 0.25 wt %). These tablets were treated with near-IR laser radiation at varying intensities and exposure time. It was demonstrated that with a higher laser intensity and PN content, the onset and rate of amorphization was faster due to the shorter exposure required to reach the temperature threshold, above which the on-demand amorphization of drugs was shown to take place. Moreover, to achieve full amorphization in the compacts with a higher drug content, a longer duration of laser irradiation was needed as compared to the compacts with lower drug content at the identical tablet thickness [88].

#### 3.3.3. Proposed Mechanism of In Situ Amorphization

From a mechanistic viewpoint, the dominant hypothesis so far is that the mobile polymers work as solvents, to dissolve and interact with the drugs. As the hygroscopic polymers absorb moisture, their molecular mobility increases, allowing them to have increased chances to be in contact with the drug particles in crystalline state, and form interactions that lead to the loss of crystallinity [93]. In the microwave/laser irradiation methods, as the drug-polymer mixture is heated, the temperature increases above the T_g_ of the polymer and the amorphization process is accelerated.

To investigate the validity of the suggested mechanism, some of the studies focused on exploring the factors affecting the rate and degree of amorphization, e.g., particle size, temperature and viscosity. These factors are all components of a well-known model that explains the dissolution kinetics of drugs: the Noyes–Whitney equation. Hempel et al., in their microwave induced amorphization studies, demonstrated that reducing the size of the particle (drug and polymer) and heating the samples to get higher temperatures (above T_g_) and lower viscosity increased the rate of the on-demand amorphization [83,90]. Thus, the kinetics of in situ glass formation and that of the drug dissolution in the polymer was shown to be correlated, which seems to play a crucial role in understanding the underlying mechanism of in situ amorphization. Simple as it may be, however, the suggested explanation of the underlying mechanism is not very clear, raising some questions such as what happens, on a molecular level, in the interval between the dissolution of the drug in the polymeric network and the amorphization, and how exactly it is causing the phase transformation. Further in-depth studies seem to be required to answer such questions.

An in situ amorphization approach comes with some apparent advantages that lie in its possibility to circumvent the physical stability issue, and the simplicity of the preparation step. However, being in its conceptual phase, the technique is faced with various challenges as the current understanding of its mechanism is yet insufficient and its applicability to broader range of drugs and excipients has not been explored, along with some other issues already mentioned in the literature [75]. Therefore, in future studies, each of these topics needs to be addressed to better understand and make full use of such an interesting and promising approach to generate amorphous drug formulations.

## 4. Conclusions and Future Perspectives

Solid dispersions have been considered as one of the most effective ways to improve the low solubility and dissolution of poorly water-soluble drugs. However, the drug incorporated in the polymer matrix largely tends to recrystallize during fabrication and storage conditions triggered by thermodynamic force. Furthermore, due to the drug solubility limitation in the polymer matrices, large amounts of polymers are required for stabilizing the amorphous drug, which thereby leads to the total volume increase in final dosage forms. To overcome these problems and provide more effective options, new approaches for amorphous formulations have been introduced such as co-amorphization, mesoporous particle-based amorphization, and in situ amorphization. Therefore, in this review, the basic concept of each approach was briefly summarized, and the recent research topics on this strategy were comprehensively discussed with adequate categorization by an extensive literature review on the latest publications in the last five years.

Other pharmaceutical techniques have been investigated to improve solubility and dissolution of poorly water-soluble drugs, such as cocrystals, salt formation, and nanopharmaceuticals. Since they are explored frequently for the same purposes with ASD, the commercialization of poorly water-soluble drugs employing the techniques would depend upon the degree of stability and solubility being improved, as well as the cost of processes or manufacturing. The cost of manufacturing will largely be affected by whether new equipment is required and the total duration of the process.

Stability has been the main issue for the commercial product development of the ASDs because of the inherent metastability driving recrystallization during preparation and storage. Thus, to solve such limitations to some extent, novel approaches have emerged, which are being studied with varying topics as thoroughly discussed in this review. Even though novel amorphization techniques such as co-amorphous systems, mesoporous particle-based amorphous systems, and in-situ amorphization are considered as promising alternatives to PASDs, more investigations need to be carried out for the large-scale industrial applications. In particular, co-amorphous systems formed with low molecular weight co-formers would be advantageous to be applicable for pharmaceutical industry, as they can efficiently be produced to smaller dosage forms. In case of in-situ amorphization, industrial-sized microwave oven or laser-emitting equipment would be required to treat optimized formulations in a factory. Evidently, the science of drug amorphization is advancing rapidly, and we expect that these current advancements will provide next generation delivery systems, especially for poorly soluble drugs.

## Figures and Tables

**Table 2:** Recent examples of co-formers used to generate co-amorphous systems.

Drug	Carrier	Preparation Method	Key Findings	Ref.
Drug-Drug Co-Amorphous Systems				
Valsartan	Nimodipine	Quench cooling	This possible combination therapy agent for hypertension was successfully formulated as a co-amorphous system.	[33]
Flutamide	Bicalutamide Bicalutamide-MMA/EA or PVP	Melt Quenching	This drug-drug co-amorphous system was successfully prepared. However, the addition of polymer was required to achieve complete inhibition of recrystallization.	[34]
Drug-Excipient Co-Amorphous Systems				
Carvedilol	L-aspartic acid L-glutamic acid	Spray drying	L-aspartic acid and L-glutamic acid were demonstrated to form co-amorphous mixtures with carvedilol at varying molar ratios	[35]
Griseofulvin	DL-methionine L-aspartic acid L-valine L-lysine Tryptophan	Ball milling	The first attempt to develop griseofulvin: An amino acid co-amorphous system. Tryptophan showed the best results in stabilizing the amorphous mixture.	[36]
6 drugs	20 amino acids	Ball milling	Co-formability between six model drugs and 20 amino acids was evaluated, which can be a guide to find a successful co-amorphous formulation.	[37]
36 drugs	Amino acid formulations	Ball milling	Different co-amorphous combinations were characterized in terms of co-formability, storage stability, and the dissolution rate.	[38]
Mebendazole	Trp-Phe Phe-Trp Asp-Tyr His-Gly Pro-Trp	Ball milling	All 5 dipeptides formed to have stable co-amorphous mixtures.	[39]
Curcumin	Piperine	Quench cooling	Increased bioavailability by inhibition of the intestinal metabolism and enhanced membrane permeability, the effect of co-forming with piperine	[40]
Carbamazepine	Citric acid-L-Arginine	Ball milling	The combination of carbamazepine-citric acid-arginine formed a stable ternary co-amorphous system, suggesting the importance of selecting a desirable salt co-former.	[41]
Various drugs	Natural bile acid sodium taurocholate	Ball milling	Natural bile acid sodium taurocholate was shown to be an effective co-former due to its unusual molecular shape that impedes recrystallization and its high capacity to form hydrogen bonding.	[42]
Mebendazole, Tadalafil, Piroxicam	Aspartame	Ball milling	Aspartame was shown to be a promising option as a co-former. It demonstrated the effectiveness of dipeptide as a co-former, having the advantages of each amino acid component.	[43]
Carvedilol	Benzoic acid Malic acid Citric acid	Spray drying	Malic acid and Citric acid, having two and three free carboxyl group respectively, could successfully generate stable co-amorphous mixtures.	[44]
Carbamazepine Indomethacin	Tannic acid	Solvent evaporation	Complete amorphization with a superior stabilizing effect	[45]

MMA/EA, methyl methacrylate-co-ethyl acrylate; PVP, Polyvinylpyrrolidone; Trp, Tryptophan; Phe, Phenylalanine; Asp, aspartic acid; His, histidine; Gly, glycine; Pro, proline.

**Table 3:** Recent publications on the predictive evaluation of co-amorphous formation.

Drug	Carrier	Preparation Method	Key Findings	Ref.
Indomethacin Carvedilol	Tryptophan	Ball milling	The glass transition temperature for β-relaxation can be employed to predict the potential of the formation of the drug-amino acids co-amorphous systems.	[46]
Naproxen Indomethacin	Cimetidine Naproxen Paracetamol Cimetidine Terfenadine	Quench cooling	A scanning electron microscope with an energy dispersive X-ray spectrometer (SEM-EDS) can be utilized to detect the early-state phase separation of small amounts of amorphous mixtures.	[47]
Carbamazepine	Organic acid Saccharin Nicotinamide	Ball milling Quench cooling	Mixtures that show a tendency to form co-crystals were not suitable for co-amorphous formulations, as they are likely to recrystallize. Co-formers with the possibility of co-crystal formation can be excluded when designing the co-amorphous formulations.	[48]
Various mixtures	Various mixtures	Melt quenching	Mixing enthalpy (ΔH_mix_) and Δlog *p* value can be a useful indicator for predicting the formation of co-amorphous systems.	[49]
6 drugs	20 amino acids	Ball milling	By analyzing multiple molecular descriptors with various potential co-amorphous combinations, this study provided a useful model for predicting the suitable excipient for the ball milling technique.	[50]
Furosemide Cimetidine Nitrofurantoin Mebendazole	L-Arginine, L-Citrulline	Ball milling	Structurally related amino acids were selected to investigate how salt formation and the structural similarity of co-formers affect the co-amorphous systems. Neither the salt forming character nor the structural similarity were the prerequisites to achieving successful co-amorphous systems.	[51]

**Table 4:** Recent examples of co-amorphous systems applicable for different routes of administration.

Drug.	Carrier	Preparation Method	Key Findings	Ref.
Atenolol	Urea	Melt quenching	The co-amorphous based supersaturated formulation was shown to significantly enhance the skin permeation of atenolol, suggesting its potential for transdermal use.	[52]
Ofloxacin	Tryptophan	Lyophilization	By generating a co-amorphous ofloxacin-tryptophan system, the solubility of ofloxacin was shown to increase tenfold, which would enable parenteral delivery at high doses.	[53]
Budesonide	Arginine	Spray drying	Spray drying was shown to be an effective method to produce inhalable co-amorphous systems.	[54]
Simvastatin	Leucine Tryptophan Lysine	Spray drying	The formation of co-amorphous systems with amino acids was demonstrated to improve the physical stability of spray-dried simvastatin for inhalation.	[55]

**Table 5:** Recent publications on the development of drug loading techniques for mesoporous particle-based amorphization.

Drug	Carrier	Preparation Method	Key Findings	Ref.
Meropenem	MCM-41	Liquid carbon dioxide method	Liquid carbon dioxide could be used to load meropenem into surface-modified MCM-41, and it achieved a considerably high drug loading capacity of over 25%.	[59]
Fenofibrate	Ordered mesoporous silica (OMS-7)	Supercritical carbon dioxide impregnation method Incipient wetness impregnation method	The supercritical carbon dioxide impregnation method achieved a higher fenofibrate loading and shorter impregnation time compared to the conventional incipient wetness method.	[60]
Clofazimine	Mesoporous silica synthesized in the laboratory	Chaperone-assisted drug loading method	The chaperone-assisted drug loading method utilized acetophenone as the chaperone for clofazimine, and it enables a high drug loading.	[61]
Heparin Ibuprofen	SBA-15	One-pot synthesis method	The one-pot synthesis process utilizing evaporation-induced self-assembly technique achieved several-fold higher drug loading and a shorter preparation time over the conventional loading method.	[62]
Ibuprofen	Mesoporous silica synthesized in the laboratory	Fluid bed hot-melt impregnation method	As a solvent-free process, the fluid bed hot-melt impregnation was demonstrated to be efficient for drug amorphization into mesoporous particles.	[63]

MCM-41, Mobil Composition of Matter No. 41; SBA-15, Santa Barbara Amorphous-15.

**Table 7:** Recent examples of in situ amorphization employing solid dosage forms.

Drug	Carrier	Preparation Method	Key Findings	Ref.
Indomethacin	Eudragit^®^ E	Immersion in aqueous buffer (pH 6.8)	Crystalline indomethacin-Eudragit^®^ E compacts were amorphized upon immersion in buffer	[76]
Naproxen Ibuprofen	Eudragit^®^ E	Immersion in water	Compacts containing crystalline naproxen, ibuprofen/Eudragit^®^ E were amorphized upon immersion in water	[77]
Indomethacin	PVP K12 PVP K17 PVP K25	Moisture conditioning and Microwave irradiation	Moisture content, energy input, and Tg affect the degree of in situ amorphization	[78,79]
Celecoxib	PVP K12	Moisture conditioning and Microwave irradiation	Higher degree of amorphization was achieved with a lower drug content.	[80]
Indomethacin Furosemide	Arginine	Immersion in acidic medium	In situ co-amorphization was achieved upon immersion in acid, suggesting the possibility of in vivo amorphization in the gastrointestinal tract	[81]
Carvedilol	Aspartic acid	Immersion in acidic medium	The technique was shown to be applicable for another co-amorphous combination.	[82]
Celecoxib	PVP	Moisture conditioning and Microwave irradiation	The smaller the particle size, the faster the amorphization rate and the higher the degree of amorphization	[83]
Celecoxib	PVP	Moisture conditioning and Microwave irradiation	Convective heating was shown to be less efficient for preparation of glass solutions in situ	[84]
Indomethacin	Soluplus^®^	Microwave irradiation without conditioning	Glycerol was suggested as an alternative source to absorb microwave radiation.	[85]
Celecoxib	PEG 3000 PEG 4000	Microwave irradiation without conditioning	Low viscosity and high temperature lead to faster amorphization.	[86]
Celecoxib	PVP K12	Microwave irradiation without conditioning	Crystal hydrates was suggested as the possible source of water.	[87]
Celecoxib	PVP K12 with plasmonic nanoparticles	Laser irradiation	Fully amorphous compact can be generated upon laser irradiation	[88]
Celecoxib	PVP-PEG	Microwave irradiation without conditioning	Molten PEG can cause tablet deformation and recrystallize upon cooling. PVP-PEG combination can be employed to generate fully amorphous and stable amorphous compacts.	[89]
Celecoxib	PVP K12 PVP K17	Moisture conditioning & Microwave irradiation	Linear relationship between the amorphization rate and the tablet temperature was established.	[90]

PVP, Polyvinylpyrrolidone; PEG, Polyethylene glycol; T_g_, glass transition temperature.

## References

[1] Kalepu S., Nekkanti V. (2015). Insoluble drug delivery strategies: Review of recent advances and business prospects. Acta Pharm. Sin. B.

[2] Kesisoglou F., Panmai S., Wu Y. (2007). Nanosizing—Oral formulation development and biopharmaceutical evaluation. Adv. Drug Deliv. Rev..

[3] Rautio J., Kumpulainen H., Heimbach T., Oliyai R., Oh D., Järvinen T., Savolainen J. (2008). Prodrugs: Design and clinical applications. Nat. Rev. Drug Discov..

[4] Serajuddin A.T. (2007). Salt formation to improve drug solubility. Adv. Drug Deliv. Rev..

[5] Subongkot T., Ngawhirunpat T. (2017). Development of a novel microemulsion for oral absorption enhancement of all-trans retinoic acid. Int. J. Nanomed..

[6] Cheow W.S., Kiew T.Y., Yang Y., Hadinoto K. (2014). Amorphization Strategy Affects the Stability and Supersaturation Profile of Amorphous Drug Nanoparticles. Mol. Pharm..

[7] Wlodarski K., Sawicki W., Paluch K., Tajber L., Grembecka M., Hawelek L., Wojnarowska Z., Grzybowska K., Talik E., Paluch M. (2014). The influence of amorphization methods on the apparent solubility and dissolution rate of tadalafil. Eur. J. Pharm. Sci..

[8] Baghel S., Cathcart H., O’Reilly N.J. (2016). Polymeric Amorphous Solid Dispersions: A Review of Amorphization, Crystallization, Stabilization, Solid-State Characterization, and Aqueous Solubilization of Biopharmaceutical Classification System Class II Drugs. J. Pharm. Sci..

[9] Liu Y., Sun C., Hao Y., Jiang T., Zheng L., Wang S. (2010). Mechanism of Dissolution Enhancement and Bioavailability of Poorly Water Soluble Celecoxib by Preparing Stable Amorphous Nanoparticles. J. Pharm. Pharm. Sci..

[10] Van Den Mooter G. (2012). The use of amorphous solid dispersions: A formulation strategy to overcome poor solubility and dissolution rate. Drug Discov. Today Technol..

[11] Serajuddin A.T. (1999). Solid dispersion of poorly water-soluble drugs: Early promises, subsequent problems, and recent breakthroughs. J. Pharm. Sci..

[12] Song Y., Wang L., Yang P., Wenslow R.M., Tan B., Zhang H., Deng Z. (2013). Physicochemical Characterization of Felodipine-Kollidon VA64 Amorphous Solid Dispersions Prepared by Hot-Melt Extrusion. J. Pharm. Sci..

[13] Ziaee A., Albadarin A.B., Padrela L., Faucher A., O’Reilly E., Walker G. (2017). Spray drying ternary amorphous solid dispersions of ibuprofen—An investigation into critical formulation and processing parameters. Eur. J. Pharm. Biopharm..

[14] Chiou W.L., Riegelman S. (1971). Pharmaceutical Applications of Solid Dispersion Systems. J. Pharm. Sci..

[15] Ghebremeskel A.N., Vemavarapu C., Lodaya M. (2006). Use of Surfactants as Plasticizers in Preparing Solid Dispersions of Poorly Soluble API: Stability Testing of Selected Solid Dispersions. Pharm. Res..

[16] Munjal M., Stodghill S.P., ElSohly M.A., Repka M.A. (2006). Polymeric systems for amorphous Δ9-tetrahydrocannabinol produced by a hot-melt method. Part I: Chemical and thermal stability during processing. J. Pharm. Sci..

[17] Zhang D., Lee Y.-C., Shabani Z., Lamm C.F., Zhu W., Li Y., Templeton A. (2018). Processing Impact on Performance of Solid Dispersions. Pharmaceutics.

[18] Kwong A.D., Kauffman R.S., Hurter P., Mueller P. (2011). Discovery and development of telaprevir: An NS3-4A protease inhibitor for treating genotype 1 chronic hepatitis C virus. Nat. Biotechnol..

[19] Zhao M., Barker S.A., Belton P.S., McGregor C., Craig D.Q. (2012). Development of fully amorphous dispersions of a low Tg drug via co-spray drying with hydrophilic polymers. Eur. J. Pharm. Biopharm..

[20] Chavan R., Thipparaboina R., Kumar D., Shastri N.R. (2016). Co amorphous systems: A product development perspective. Int. J. Pharm..

[21] Chieng N., Aaltonen J., Saville D., Rades T. (2009). Physical characterization and stability of amorphous indomethacin and ranitidine hydrochloride binary systems prepared by mechanical activation. Eur. J. Pharm. Biopharm..

[22] Löbmann K., Grohganz H., Laitinen R., Strachan C., Rades T. (2013). Amino acids as co-amorphous stabilizers for poorly water soluble drugs—Part 1: Preparation, stability and dissolution enhancement. Eur. J. Pharm. Biopharm..

[23] Allesø M., Chieng N., Rehder S., Rantanen J., Rades T., Aaltonen J. (2009). Enhanced dissolution rate and synchronized release of drugs in binary systems through formulation: Amorphous naproxen–cimetidine mixtures prepared by mechanical activation. J. Control. Release.

[24] Dengale S.J., Hussen S.S., Krishna B., Musmade P.B., Shenoy G.G., Bhat K. (2015). Fabrication, solid state characterization and bioavailability assessment of stable binary amorphous phases of Ritonavir with Quercetin. Eur. J. Pharm. Biopharm..

[25] Löbmann K., Laitinen R., Grohganz H., Gordon K., Strachan C., Rades T. (2011). Coamorphous Drug Systems: Enhanced Physical Stability and Dissolution Rate of Indomethacin and Naproxen. Mol. Pharm..

[26] Ueda H., Muranushi N., Sakuma S., Ida Y., Endoh T., Kadota K., Tozuka Y. (2016). A Strategy for Co-former Selection to Design Stable Co-amorphous Formations Based on Physicochemical Properties of Non-steroidal Inflammatory Drugs. Pharm. Res..

[27] Yu L., Reutzel-Edens A.S.M., Mitchell C.A. (2000). Crystallization and Polymorphism of Conformationally Flexible Molecules: Problems, Patterns, and Strategies. Org. Process. Res. Dev..

[28] Slámová M., Prausová K., Epikaridisová J., Brokešová J., Kuentz M., Patera J., Zámostný P. (2021). Effect of co-milling on dissolution rate of poorly soluble drugs. Int. J. Pharm..

[29] Moinuddin S.M., Ruan S., Huang Y., Gao Q., Shi Q., Cai B., Cai T. (2017). Facile formation of co-amorphous atenolol and hydrochlorothiazide mixtures via cryogenic-milling: Enhanced physical stability, dissolution and pharmacokinetic profile. Int. J. Pharm..

[30] Mishra J., Löbmann K., Grohganz H., Rades T. (2018). Influence of preparation technique on co-amorphization of carvedilol with acidic amino acids. Int. J. Pharm..

[31] D’Angelo A., Edgar B., Hurt A.P., Antonijević M.D. (2018). Physico-chemical characterisation of three-component co-amorphous systems generated by a melt-quench method. J. Therm. Anal. Calorim..

[32] Park H., Seo H.J., Hong S.-H., Ha E.-S., Lee S., Kim J.-S., Baek I.-H., Kim M.-S., Hwang S.-J. (2020). Characterization and therapeutic efficacy evaluation of glimepiride and L-arginine co-amorphous formulation prepared by supercritical antisolvent process: Influence of molar ratio and preparation methods. Int. J. Pharm..

[33] Lodagekar A., Chavan R.B., Mannava M.K.C., Yadav B., Chella N., Nangia A.K., Shastri N.R. (2019). Co amorphous valsartan nifedipine system: Preparation, characterization, in vitro and in vivo evaluation. Eur. J. Pharm. Sci..

[34] Pacult J., Rams-Baron M., Chmiel K., Jurkiewicz K., Antosik A., Szafraniec J., Kurek M., Jachowicz R., Paluch M. (2019). How can we improve the physical stability of co-amorphous system containing flutamide and bicalutamide? The case of ternary amorphous solid dispersions. Eur. J. Pharm. Sci..

[35] Liu J., Rades T., Grohganz H. (2020). Determination of the Optimal Molar Ratio in Amino Acid-Based Coamorphous Systems. Mol. Pharm..

[36] França M.T., Marcos T.M., Pereira R.N., Stulzer H.K. (2020). Could the small molecules such as amino acids improve aqueous solubility and stabilize amorphous systems containing Griseofulvin?. Eur. J. Pharm. Sci..

[37] Kasten G., Grohganz H., Rades T., Löbmann K. (2016). Development of a screening method for co-amorphous formulations of drugs and amino acids. Eur. J. Pharm. Sci..

[38] Kasten G., Löbmann K., Grohganz H., Rades T. (2019). Co-former selection for co-amorphous drug-amino acid formulations. Int. J. Pharm..

[39] Wu W., Löbmann K., Schnitzkewitz J., Knuhtsen A., Pedersen D.S., Rades T., Grohganz H. (2018). Dipeptides as co-formers in co-amorphous systems. Eur. J. Pharm. Biopharm..

[40] Wang R., Han J., Jiang A., Huang R., Fu T., Wang L., Zheng Q., Li W., Li J. (2019). Involvement of metabolism-permeability in enhancing the oral bioavailability of curcumin in excipient-free solid dispersions co-formed with piperine. Int. J. Pharm..

[41] Ueda H., Wu W., Löbmann K., Grohganz H., Müllertz A., Rades T. (2018). Application of a Salt Coformer in a Co-Amorphous Drug System Dramatically Enhances the Glass Transition Temperature: A Case Study of the Ternary System Carbamazepine, Citric Acid, and l-Arginine. Mol. Pharm..

[42] Gniado K., MacFhionnghaile P., McArdle P., Erxleben A. (2018). The natural bile acid surfactant sodium taurocholate (NaTC) as a coformer in coamorphous systems: Enhanced physical stability and dissolution behavior of coamorphous drug-NaTc systems. Int. J. Pharm..

[43] Wu W., Löbmann K., Schnitzkewitz J., Knuhtsen A., Pedersen D.S., Grohganz H., Rades T. (2018). Aspartame as a co-former in co-amorphous systems. Int. J. Pharm..

[44] Wu W., Ueda H., Löbmann K., Rades T., Grohganz H. (2018). Organic acids as co-formers for co-amorphous systems—Influence of variation in molar ratio on the physicochemical properties of the co-amorphous systems. Eur. J. Pharm. Biopharm..

[45] Fael H., Demirel A.L. (2020). Tannic acid as a co-former in co-amorphous systems: Enhancing their physical stability, solubility and dissolution behavior. Int. J. Pharm..

[46] Kissi E.O., Kasten G., Löbmann K., Rades T., Grohganz H., Loebmann K. (2018). The Role of Glass Transition Temperatures in Coamorphous Drug–Amino Acid Formulations. Mol. Pharm..

[47] Pajula K., Hyyryläinen J., Koistinen A., Leskinen J.T., Korhonen O. (2020). Detection of amorphous-amorphous phase separation in small molecular co-amorphous mixtures with SEM-EDS. Eur. J. Pharm. Biopharm..

[48] Wu W., Wang Y., Löbmann K., Grohganz H., Rades T. (2019). Transformations between Co-Amorphous and Co-Crystal Systems and Their Influence on the Formation and Physical Stability of Co-Amorphous Systems. Mol. Pharm..

[49] Mizoguchi R., Waraya H., Hirakura Y. (2019). Application of Co-Amorphous Technology for Improving the Physicochemical Properties of Amorphous Formulations. Mol. Pharm..

[50] Meng-Lund H.M.L., Kasten G., Jensen K.T., Poso A., Pantsar T., Rades T., Rantanen J., Grohganz H. (2018). The use of molecular descriptors in the development of co-amorphous formulations. Eur. J. Pharm. Sci..

[51] Wu W., Löbmann K., Rades T., Grohganz H. (2018). On the role of salt formation and structural similarity of co-formers in co-amorphous drug delivery systems. Int. J. Pharm..

[52] Hirakawa Y., Ueda H., Miyano T., Kamiya N., Goto M. (2019). New insight into transdermal drug delivery with supersaturated formulation based on co-amorphous system. Int. J. Pharm..

[53] Zhu S., Gao H., Babu S., Garad S. (2018). Co-Amorphous Formation of High-Dose Zwitterionic Compounds with Amino Acids To Improve Solubility and Enable Parenteral Delivery. Mol. Pharm..

[54] Lu W., Rades T., Rantanen J., Yang M. (2019). Inhalable co-amorphous budesonide-arginine dry powders prepared by spray drying. Int. J. Pharm..

[55] Lu W., Rades T., Rantanen J., Chan H.-K., Yang M. (2019). Amino acids as stabilizers for spray-dried simvastatin powder for inhalation. Int. J. Pharm..

[56] Li Z., Zhang Y., Feng N. (2019). Mesoporous silica nanoparticles: Synthesis, classification, drug loading, pharmacokinetics, biocompatibility, and application in drug delivery. Expert Opin. Drug Deliv..

[57] Vallet-Regí M., Balas F., Arcos D. (2007). Mesoporous Materials for Drug Delivery. Angew. Chem. Int. Ed..

[58] Maleki A., Kettiger H., Schoubben A., Rosenholm J.M., Ambrogi V., Hamidi M. (2017). Mesoporous silica materials: From physico-chemical properties to enhanced dissolution of poorly water-soluble drugs. J. Control. Release.

[59] Raza A., Sime F.B., Cabot P.J., Roberts J.A., Falconer J.R., Kumeria T., Popat A. (2021). Liquid CO2 Formulated Mesoporous Silica Nanoparticles for pH-Responsive Oral Delivery of Meropenem. ACS Biomater. Sci. Eng..

[60] Bouledjouidja A., Masmoudi Y., Van Speybroeck M., Schueller L., Badens E. (2016). Impregnation of Fenofibrate on mesoporous silica using supercritical carbon dioxide. Int. J. Pharm..

[61] Chen W., Cheng C.-A., Lee B.-Y., Clemens D.L., Huang W.-Y., Horwitz M.A., Zink J.I. (2018). Facile Strategy Enabling Both High Loading and High Release Amounts of the Water-Insoluble Drug Clofazimine Using Mesoporous Silica Nanoparticles. ACS Appl. Mater. Interfaces.

[62] Wan M.M., Li Y.Y., Yang T., Zhang T., Sun X.D., Zhu J.H. (2016). In Situ Loading of Drugs into Mesoporous Silica SBA-15. Chem. A Eur. J..

[63] Mužík J., Lizoňová D., Zadražil A., Štěpánek F. (2020). Drug amorphisation by fluid bed hot-melt impregnation of mesoporous silica carriers. Chem. Eng. J..

[64] Szegedi Á, Shestakova P., Trendafilova I., Mihayi J., Tsacheva I., Mitova V., Kyulavska M., Koseva N., Momekova D., Konstantinov S. (2019). Modified mesoporous silica nanoparticles coated by polymer complex as novel curcumin delivery carriers. J. Drug Deliv. Sci. Technol..

[65] Lang Y., Finn D., Pandit A., Walsh P. (2011). Pharmacological activity of ibuprofen released from mesoporous silica. J. Mater. Sci. Mater. Med..

[66] Narayan R., Gadag S., Cheruku S.P., Raichur A.M., Day C.M., Garg S., Manandhar S., Pai K.S.R., Suresh A., Mehta C.H. (2021). Chitosan-glucuronic acid conjugate coated mesoporous silica nanoparticles: A smart pH-responsive and receptor-targeted system for colorectal cancer therapy. Carbohydr. Polym..

[67] Li-Hong W., Xin C., Hui X., Li-Li Z., Jing H., Mei-Juan Z., Jie L., Yi L., Jin-Wen L., Wei Z. (2013). A novel strategy to design sustained-release poorly water-soluble drug mesoporous silica microparticles based on supercritical fluid technique. Int. J. Pharm..

[68] Limnell T., Santos H.A., Mäkilä E., Heikkilä T., Salonen J., Murzin D., Kumar N., Laaksonen T., Peltonen L., Hirvonen J.T. (2011). Drug Delivery Formulations of Ordered and Nonordered Mesoporous Silica: Comparison of Three Drug Loading Methods. J. Pharm. Sci..

[69] Tourne-Peteilh C., Begu S., Lerner D.A., Galarneau A., Lafont U., Devoisselle J.-M. (2011). Sol–gel one-pot synthesis in soft conditions of mesoporous silica materials ready for drug delivery system. J. Sol-Gel Sci. Technol..

[70] Ayad M.M., Salahuddin N.A., Abu El-Nasr A., Torad N. (2016). Amine-functionalized mesoporous silica KIT-6 as a controlled release drug delivery carrier. Microporous Mesoporous Mater..

[71] Khosraviyan P., Ardestani M.S., Khoobi M., Ostad S.N., Dorkoosh F.A., Javar H.A., Amanlou M. (2016). Mesoporous silica nanoparticles functionalized with folic acid/methionine for active targeted delivery of docetaxel. OncoTargets Ther..

[72] Ortiz-Islas E., Sosa-Arróniz A., Manríquez-Ramírez M.E., Rodríguez-Pérez C.E., Tzompantzi F., Padilla J.M. (2021). Mesoporous silica nanoparticles functionalized with folic acid for targeted release Cis-Pt to glioblastoma cells. Rev. Adv. Mater. Sci..

[73] Asgari M., Soleymani M., Miri T., Barati A. (2021). Design of thermosensitive polymer-coated magnetic mesoporous silica nanocomposites with a core-shell-shell structure as a magnetic/temperature dual-responsive drug delivery vehicle. Polym. Adv. Technol..

[74] Priemel P.A., Grohganz H., Rades T. (2016). Unintended and in situ amorphisation of pharmaceuticals. Adv. Drug Deliv. Rev..

[75] Qiang W., Löbmann K., McCoy C., Andrews G., Zhao M. (2020). Microwave-Induced In Situ Amorphization: A New Strategy for Tackling the Stability Issue of Amorphous Solid Dispersions. Pharmaceutics.

[76] Priemel P.A., Laitinen R., Grohganz H., Rades T., Strachan C. (2013). In situ amorphisation of indomethacin with Eudragit^®^ E during dissolution. Eur. J. Pharm. Biopharm..

[77] Doreth M., Löbmann K., Grohganz H., Holm R., de Diego H.L., Rades T., Priemel P.A. (2016). Glass solution formation in water—In situ amorphization of naproxen and ibuprofen with Eudragit^®^ E PO. J. Drug Deliv. Sci. Technol..

[78] Doreth M., Hussein M.A., Priemel P.A., Grohganz H., Holm R., de Diego H.L., Rades T., Löbmann K. (2017). Amorphization within the tablet: Using microwave irradiation to form a glass solution in situ. Int. J. Pharm..

[79] Doreth M., Löbmann K., Priemel P., Grohganz H., Taylor R., Holm R., de Diego H.L., Rades T. (2018). Influence of PVP molecular weight on the microwave assisted in situ amorphization of indomethacin. Eur. J. Pharm. Biopharm..

[80] Edinger M., Knopp M.M., Kerdoncuff H., Rantanen J., Rades T., Löbmann K. (2018). Quantification of microwave-induced amorphization of celecoxib in PVP tablets using transmission Raman spectroscopy. Eur. J. Pharm. Sci..

[81] Petry I., Löbmann K., Grohganz H., Rades T., Leopold C.S. (2018). In situ co-amorphisation of arginine with indomethacin or furosemide during immersion in an acidic medium—A proof of concept study. Eur. J. Pharm. Biopharm..

[82] Petry I., Löbmann K., Grohganz H., Rades T., Leopold C.S. (2019). In situ co-amorphisation in coated tablets—The combination of carvedilol with aspartic acid during immersion in an acidic medium. Int. J. Pharm..

[83] Hempel N., Knopp M.M., Berthelsen R., Zeitler J.A., Löbmann K. (2020). The influence of drug and polymer particle size on the in situ amorphization using microwave irradiation. Eur. J. Pharm. Biopharm..

[84] Hempel N.-J., Knopp M.M., Berthelsen R., Löbmann K. (2020). Convection-Induced vs. Microwave Radiation-Induced in situ Drug Amorphization. Molecules.

[85] Hempel N.-J., Morsch F., Knopp M.M., Berthelsen R., Löbmann K. (2021). The Use of Glycerol as an Enabling Excipient for Microwave-Induced In Situ Drug Amorphization. J. Pharm. Sci..

[86] Hempel N.-J., Dao T., Knopp M.M., Berthelsen R., Löbmann K. (2020). The Influence of Temperature and Viscosity of Polyethylene Glycol on the Rate of Microwave-Induced In Situ Amorphization of Celecoxib. Molecules.

[87] Holm T.P., Knopp M.M., Löbmann K., Berthelsen R. (2021). Microwave induced in situ amorphisation facilitated by crystalline hydrates. Eur. J. Pharm. Sci..

[88] Hempel N.-J., Merkl P., Asad S., Knopp M.M., Berthelsen R., Bergström C.A., Teleki A., Sotiriou G.A., Löbmann K. (2021). Utilizing Laser Activation of Photothermal Plasmonic Nanoparticles to Induce On-Demand Drug Amorphization inside a Tablet. Mol. Pharm..

[89] Hempel N.-J., Knopp M., Löbmann K., Berthelsen R. (2021). Studying the Impact of the Temperature and Sorbed Water during Microwave-Induced In Situ Amorphization: A Case Study of Celecoxib and Polyvinylpyrrolidone. Pharmaceutics.

[90] Hempel N.-J., Knopp M.M., Zeitler J.A., Berthelsen R., Löbmann K. (2021). Microwave-Induced in Situ Drug Amorphization Using a Mixture of Polyethylene Glycol and Polyvinylpyrrolidone. J. Pharm. Sci..

[91] Petry I., Löbmann K., Grohganz H., Rades T., Leopold C.S. (2018). Undesired co-amorphisation of indomethacin and arginine during combined storage at high humidity conditions. Int. J. Pharm..

[92] Bonde M.N., Sohani A.C., Daud A.S., Sapkal N.P. (2011). Microwave: An emerging trend in pharmaceutical processes and formulations. Int. J. Pharm. Technol..

[93] Marsac P.J., Romary D.P., Shamblin S.L., Baird J.A., Taylor L.S. (2008). Spontaneous Crystallinity Loss of Drugs in the Disordered Regions of Poly(Ethylene Oxide) in the Presence of Water. J. Pharm. Sci..

